# Socioeconomic Urban Environment in Latin America: Towards a Typology of Cities

**DOI:** 10.3390/su15086380

**Published:** 2023-04-07

**Authors:** Gervásio F. dos Santos, Alejandra Vives Vergara, Mauricio Fuentes-Alburquenque, José Firmino de Sousa Filho, Aureliano Sancho Paiva, Andres Felipe Useche, Goro Yamada, Tania Alfaro, Amélia A. Lima Friche, Roberto F. S. Andrade, Maurício L. Barreto, Waleska Teixeira Caiaffa, Ana V. Diez-Roux

**Affiliations:** 1 Center of Data and Knowledge Integration for Health (CIDACS, Fio Cruz—BA), Faculty of Economics, Federal University of Bahia, Salvador 40170-110, Brazil; 2 Department of Public Health, School of Medicine, CEDEUS Pontificia Universidad Católica de Chile, Santiago 8331150, Chile; 3 School of Public Health, University of Chile, Santiago 8380000, Chile; 4 Department of Industrial Engineering, Faculty of Engineering, Universidad de los Andes, Bogota 111711, Colombia; 5 Dornsife School of Public Health, Drexel University, Philadelphia, PA 19104, USA; 6 Observatory for Urban Health in Belo Horizonte (OSUBH), Speech, Language and Hearing Sciences Department, School of Medicine, Federal University of Minas Gerais, Belo Horizonte 31270-901, Brazil

**Keywords:** socioeconomic typology of cities, Latin America, finite mixture models

## Abstract

This paper aims to identify typologies of Latin American cities based on socioeconomic urban environment patterns. We used census data from 371 urban agglomerations in 11 countries included in the SALURBAL project to identify socioeconomic typologies of cities in Latin America. Exploratory factor analysis was used to select a set of variables, and finite mixture modelling (FMM) was applied to identify clusters to define the typology of cities. Despite the heterogeneities among the Latin American cities, we also found similarities. By exploring intersections and contrasts among these clusters, it was possible to define five socioeconomic regional typology patterns. The main features of each one are low-education cities in Northeast Brazil; low-unemployment cities in Peru and Panama; high-education cities in Argentina, Chile, Colombia, Costa Rica, Nicaragua and Mexico; high female labor participation, with high primary education in Argentina and low primary education in Brazil; and low female labor participation and low education in Brazil, Colombia, El Salvador, Guatemala, and Mexico. Identifying clusters of cities with similar features underscores understanding of the urban social and economic development dynamics and assists in studying how urban features affect health, the environment, and sustainability.

## 1 Introduction

The formation of cities results from dynamic processes involving economic, social, and demographic trends interacting with geography and space [1–4]. Cities worldwide share many standard features, including high population density, industrialisation, and socioeconomic inequalities [5–7]. They are also integral parts of social and economic networks related to the global dynamics of production, work, and geopolitics [8,9].

We consider cities as complex systems with specific social, environmental, and economic features that can be represented by sets of interrelated indicators [10,11]. The clustering of these features can result in typologies that can be useful in understanding urban environments and their links to quality-of-life outcomes, including health, education, income, employment, and others. Identifying clusters of cities with similar features may also facilitate the implementation of policies capable of improving the sustainability and health of these cities.

Prior European studies used several clustering methods to create groups or typologies of cities based on multiple social and economic indicators. Cluster analysis with parameterised Gaussian mixtures models was used to develop a typology of metropolitan regions in Australia. They identified 7 clusters that differed in socio-spatial structures, industry and labor relations, and patterns of economic development [9]. Other studies used the k-means method to classify 385 European cities into 10 clusters using 59 economic, social, and environmental indicators from different domains. Clusters often include cities from the same country or region. One cluster included cities in Spain and another in Eastern Europe [12]. In addition, two clusters had just one city each (Paris and London), illustrating how large cities have unique characteristics.

Few studies have investigated the heterogeneity of cities or identified clusters or typologies of cities in Latin America. Khan and Zerby [13] used theWroclaw taxonomic method to identify clusters among 24 Latin American countries. They concluded that there is a vast disparity among countries regarding social and economic development indicators. They also reported solid socioeconomic interdependence between the studied countries, as the growth of one country is usually related to the growth of others in the region. However, given the high degree of urbanization, in Latin America, the differences and interdependencies of macros cities or urban agglomerations in the region have yet to be better characterized. Although Latin American cities share many common features, they are also heterogeneous. Studies have investigated the complexity of large urban agglomerations in the region and its implications for sustainability [14]. Others have reported how globalisation has impacted Latin America differently from other world regions [15].

Globalisation has intensified the migration of the labor force to the service sector in Latin America, which has increased informality in employment with the associated low wages and lack of social protection [16,17]. From 2014 to 2019, the GDP in Latin America and the Caribbean rose, on average, 0.3% per year, while the poverty rate increased from 7.8% to 11.3% [18]. As one of the most urbanised developing regions in the world, Latin America has 80% of the 500 million people living in cities [19]. Well-managed urbanisation can maximise the benefits of high levels of population density, minimising environmental degradation and increasing sustainability [20,21]. Understanding whether cities can be clustered into types based on social and economic indicators may help understanding of the historical dynamics shaping city social and economic development and can also be useful in relating city features to health and environmental outcomes including sustainability outcomes.

Given the high levels of urbanization in the region and the relevance of social and economic processes in cities, this paper aims to identify typologies of Latin American cities based on socioeconomic urban environment patterns. Our hypothesis is that socioeconomic typologies of cities can be identified by using empirical analysis applied in a set of shared socioeconomic census-derived indicators for Latin American cities. The advantages of urbanisation have been discussed in many international forums. However, social disparities, lack of equity in income distribution, female insertion in the labor market, and the possible benefits of urbanisation still seem far from the reality of Latin American cities. Despite recent advances in combating inequalities, it continues as a relevant challenge for public policies, given the hitch governments have in maintaining social income policies capable of curbing the processes of social reproduction of poverty [22]. Therefore, this research is strongly motivated by the necessity to find socioeconomic relationships between cities in Latin America, so that policies can be designed jointly to reduce inequalities and promote sustainable development in the region.

We consider the socioeconomic environment as that formed by economic and social conditions in which communities of individuals live, work, meet their basic needs and develop economically, and build their social and family relationships. For an ecological analysis, this socioeconomic environment was captured using standardised variables from demographic census data available for 371 cities in 11 countries in Latin America with more than 100,000 inhabitants and contiguous boundaries between urban areas [23]. Therefore, our study does not consider the physical areas or volumes of the urban built-up area, or volume of activities of each city in which the socioeconomics relations happen. Scale issues are also separate from the objectives of this research once we find the socioeconomic typology of cities independent of the size of these cities.

Considering local differences, these joint conditions could generate more efficient solutions to plan cities and improve the population’s health. Although each city and country has its own characteristics, generating a city typology could contribute to identifying common policy needs, which can facilitate the multidimensional approach and strengthen international cooperation needed to aim for equitable, inclusive, and sustainable development in the continent, according to the requirements of 2030 Agenda for Sustainable Development [24].

## 2 Materials and Methods

### 2.1 Data

The SALURBAL project compiled data on the physical and social environment of cities with 100,000 residents across 11 countries of Latin America: Argentina (AR), Brazil (BR), Chile (CL), Colombia (CO), Costa Rica (CR), El Salvador (SV), Guatemala (GT), Mexico (MX), Nicaragua (NI), Panama (PA), and Peru (PE) [23]. We defined cities as urban agglomerations of administrative units (such as municipios, condados, and departamentos) that encompassed the visually apparent built-up areas of the city based on satellite imagery. Cities can include a single administrative unit (e.g., municipality) or a combination of adjacent administrative units (e.g., several municipalities) that are part of an urban extent [25].

Data on the socioeconomic environment of the cities were derived from harmonised census data using IPUMs (Integrated Public Use Microdata Series) harmonisation definitions [25]. Table A1 in Appendix A presents the constructs, variables, and data availability for these cities of 11 countries by census years. We used the following census data: Argentina (2010), Brazil (2010), Chile (2017), Colombia (2005), Costa Rica (2011), El Salvador (2007), Guatemala (2002), Mexico (2010), Nicaragua (2005), Panama (2010), and Peru (2017).

The data included 34 variables relevant for describing the social environment of cities, which were then classified into seven subdimensions grouped into three dimensions. To include all cities in the dataset, we considered only those census indicators with available data for all 371 cities (see Appendix B). It forced us to select 23 out of the 34 distinct variables, with the consequence that two out of the subdimensions listed in Table 1 (Living conditions and School attending) are not represented in this selection. One subdimension (Sanitary conditions) is characterised by just one variable. The five subdimensions that went into the final models are (i) sanitary conditions; (ii) materiality; (iii) unemployment; (iv) labor participation; and (v) education attainment. It is important to highlight that unemployment and labor participation are subdimensions that represent different aspects of the labor market dimension. The former represents the rates (%) of total male and female population 15 years or above who are unemployed. The latter represents participation in the labor force overall and by gender. Although related, these dimensions are not identical (not all persons who are not participating in the labor force are seeking employment).

### 2.2 Methodology

Exploratory Factor Analysis (EFA) was carried out to reduce the dimensionality of the available variables, grouping them into a small number of factors. We used the Kaiser–Meyer–Olkin (KMO) test to determine whether the data were suitable for applying the EFA [26]. We found a KMO = 0.66, indicating that EFA use is acceptable.

The standard EFA allows for identifying and examining groups of intercorrelated variables based on their relationship to common underlying factors. The goal is to determine the most important relationships between all observed variables and a reduced number of factors. These factors are extracted from correlation matrices using eigenvectors that represent the amount of variance each factor accounts for. Each extracted factor is associated with an eigenvalue and the corresponding eigenvector, and the first extracted factor is the one that explains the largest amount of common variance. Thus, successive eigenvalues will be smaller than the previous ones.

Based on the obtained eigenvalues, the method identified five common factors, for which the sum of proportions of common variance explained was about 86%. The corresponding values of the eigenvector components associated with these factors allowed for organising the variables into six sets, as indicated in Appendix C. They can be described in terms of their relation to the five subdimensions in Appendix A, Table A1. The factors were named according to the subdimensions most represented in each factor. These are the cases of the sets Labor Force Participation, Factor 1; Unemployment, Factor 4; and Dwelling materiality, Factor 5.

Further, the subdimension Education attainment variables were cast into two sets: Primary education, Factor 2; and Secondary and higher education, also Factor 2. Finally, added Factor 6, consisting of only the variable “Proportion of dwellings with water from a public network,” was the solely selected variable from the subdimension Sanitary condition. This subdimension was not well represented in any of the previous five factors.

Given the high correlation among the variables within sets with more than one variable, we selected just one variable to represent that construct (in the bold letter in Table A3 and the respective descriptive statistics in Table A4 in Appendix C). To determine the set variable, the criterion of highest communality and the factor loading found in a particular variable, corresponds to the adopted choice to 2 sets (Unemployment and Dwelling materiality). For the sets Labor Force Participation, Secondary and Higher Education, and Primary Education, this criterion indicated a variable associated with a specific gender. However, given the similarity of the communality statistics for unspecific and gender-specific variables, we selected the variable which was not distinguished by gender. In addition, we chose Secondary Education rather than university education based on distributions across the region. For the sanitary conditions set, we used the only variable available in that set.

### 2.3 Finite Mixture Modeling

Finite Mixture Modeling (FMM) was used to identify the socioeconomic typologies of cities. The FMM allows for identifying the probabilistic clustering that best reflects the multidimensional data structure. For details, see [22–24,27,28].

The method is based on the principle that populations can be divided into groups or subpopulations. In the absence of a variable that allows the identification of the groups, FMM can be applied to model the probability of belonging to unobserved groups. These groups represent latent classes or clusters of a specific population. The number of latent classes that best fits the data can be obtained by comparing models with different numbers of latent classes and different sets of constraints on parameters. Furthermore, the proportion of the population in each latent class can be used to predict the probability that the sample’s observations belong to each latent class.

The method accounts for interactions between indicators and provides a model-based clustering estimation. Thus, FMMallows for decomposing a dataset’s empirical distribution into a mixture of a certain number of distributions (parametric or non-parametric). For cluster analysis, it is assumed that each underlying group (or cluster) corresponds to each component of the mix of distributions.

The FMM method requires the identification of several k optimal clusters. Following [29,30], we used AIC (Akaike information criterion), BIC (Bayesian information criterion), and ICL (Integrated Complete-Data Likelihood) indices to define the k number for different values of minimum prior probabilities: 0.0; 0.05; 0.10; 0.15; and 0.20. The tests were run with specifications ranging from 1 to 10 clusters and 20 replicates for each set of minimum prior probability and the number of clusters.

We conducted the estimation process using the Estimation-Maximisation (EM) algorithm. An iterative procedure is used to obtain the posterior probabilities of data belonging to the components from prior probabilities and parameters of the distributions estimated in the previous step, with which it calculates new prior probabilities. The method uses these prior probabilities to maximise the likelihood function and estimate the component distributions’ parameters. It is repeated until we reach convergence.

## 3 Results

Table 1 shows that, for a minimum prior probability equal to 0.0, the AIC test converges once the maximum number of clusters is ten, while the BIC and ICL converge at seven clusters. However, when the minimum prior probability is 0.10, the three criteria converge at six clusters and continue converging as the minimum prior probability is increased. These results suggest that the maximal number of clusters should be k = 6.

**Figure 1:**
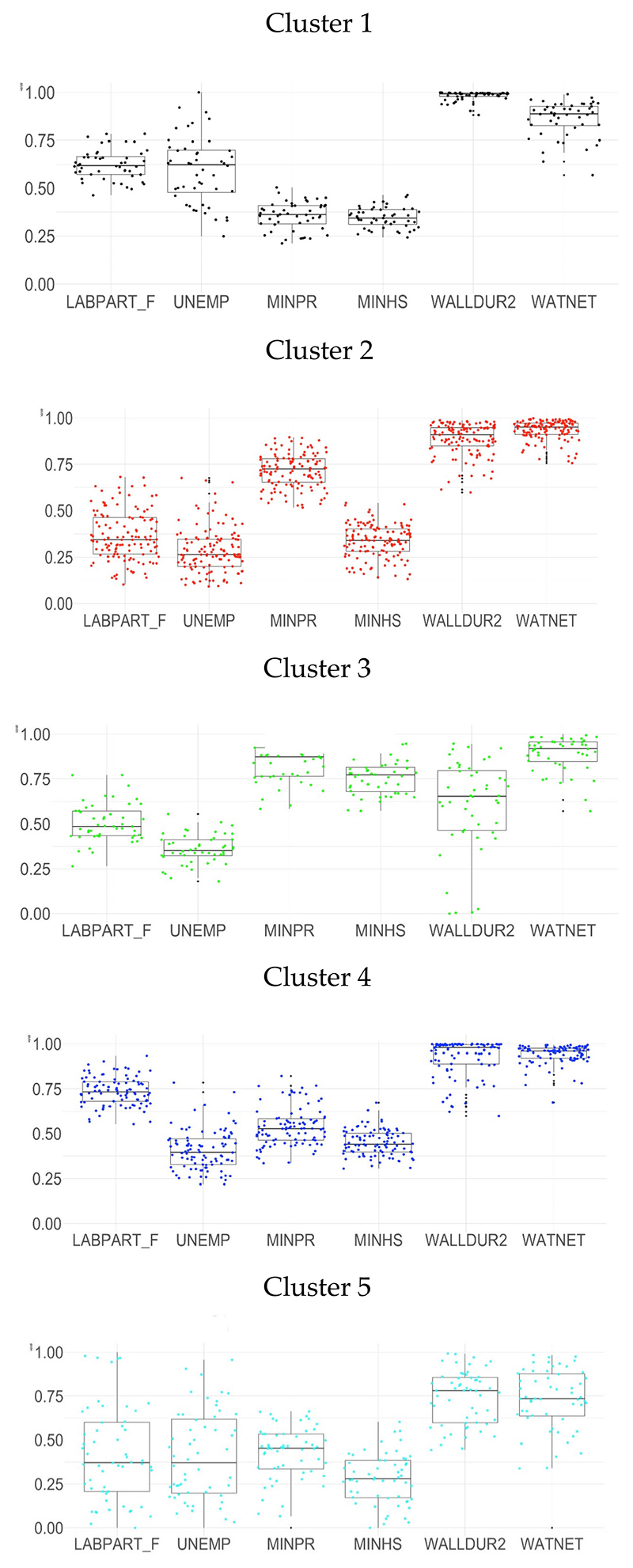
Normalised distribution of values of variables in the clusters. LABPART_F: Labor force participation rate among the female population 15 years or above; UNEMP: The unemployment rate among the total population 15 years or above in the labor force; MINPR: Proportion of the population aged 25 or older who completed primary education or above; MINHS: Proportion of the population aged 25 or older who completed secondary education or above; WALLDUR2: Proportion of dwellings with exterior walls mostly made of brick, stone, concrete, cement, and/or similar materials; WATNET: Proportion of dwellings with water from a public network.

The FMM results showed that convergence was achieved with five clusters. Figure 1 shows the statistical distribution of the six indicators for the cities grouped within each cluster. Most cities presented high proportions of the variable proportion of dwellings with water from a public network (WATNET) and the proportion of dwellings with exterior walls mostly made of brick, stone, concrete, cement, or similar materials (WALLDUR2), with exceptions for the cities of Cluster 3 (low proportion for WALLDUR2), and the cities of Cluster 5 (low proportion both variables). The distribution patterns of the other four variables are across differentiation of clusters representing socioeconomic typologies of cities.

We named each cluster according to those features that made them more distinguishable from the rest and described each cluster according to the distribution of the clustering variables.

### Cluster 1 has higher female labor force participation, higher levels of unemployment, and lower education levels

This cluster comprises 48 cities in the Northeast of Brazil and the state of Minas Gerais in the Southeast. Comparatively, the main characteristics of these cities are high levels of female labor force participation, high levels of unemployment, low primary and secondary educational levels, and a high proportion of dwellings with walls made of durable materials and with water from a public network (good housing conditions).

### Cluster 2 has lower female labor force participation, lower levels of unemployment, and higher levels of primary education

It is the largest cluster, formed by 125 cities in Argentina, Colombia, Costa Rica, Nicaragua, and Mexico. They exhibit low levels of female labor force participation and the lowest levels of unemployment. These cities reached high coverage of completed primary education but low secondary education levels, a high proportion of dwellings with walls made of durable materials, and a very high proportion with water from a public network (good housing conditions).

### Cluster 3 has moderate female labor participation, moderate levels of unemployment, and higher educational levels

This cluster is formed by 48 cities, representing all the cities in the sample for Chile, Peru, and Panama. They exhibit medium female labor force participation and unemployment rates, high primary level education and the highest levels of completed secondary education, the lowest proportion of dwellings with exterior walls made of durable materials, and a slightly lower proportion connected to a public network (fair housing conditions).

### Cluster 4 has higher female labor force participation, moderate levels of unemployment and lower educational levels

This cluster comprises 94 cities, mainly located in Southeastern/South Brazil and Argentina. It includes cities with high female participation in the labor market, medium unemployment rates as in cluster 3, low primary and secondary education coverage, and a very high proportion of dwellings with walls made of durable materials and water from a public network (good housing conditions).

### Cluster 5 has lower female labor force participation, moderate levels of unemployment, low levels of education, and poor housing conditions

This cluster is formed by 56 cities in Brazil, Colombia, El Salvador, Guatemala, and Mexico. It has a low female labor force participation, medium unemployment rates as in clusters 3 and 4, low coverage of primary and the lowest level of secondary education, as well a low proportion of dwellings with exterior walls made of durable materials, and the lowest proportion connected to a public network (poor housing conditions).

**Figure 2:**
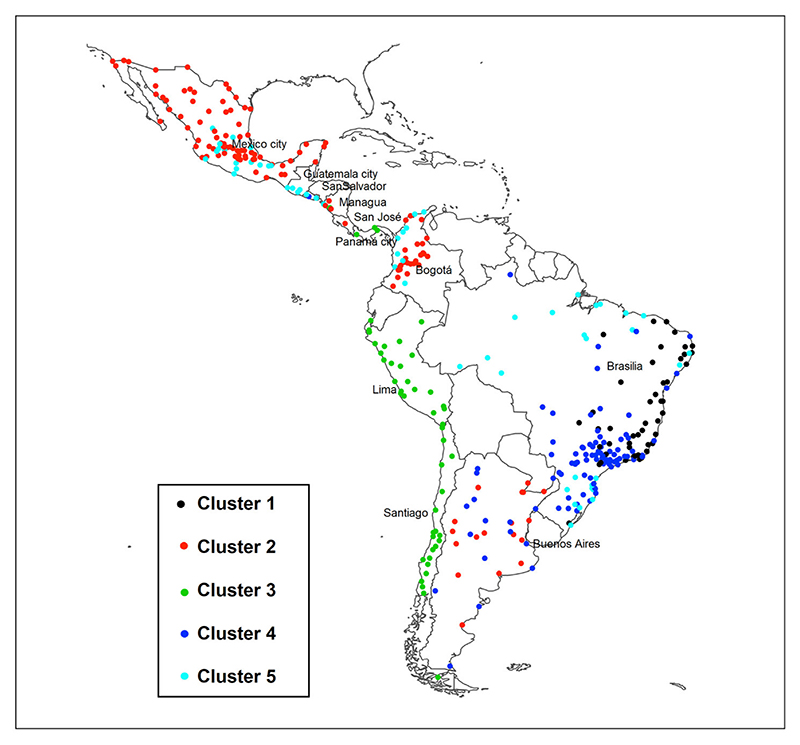
Geographic distribution of cities according to the social environment typology. See also Table A2 in Appendix B lists all cities included in the study.

Figure 2 shows the geographic location of cities in each cluster. Brazil is the country with the most significant number of cities and the most heterogeneity across cities, encompassing three types of clusters. Argentina, Colombia, Nicaragua, El Salvador, and Mexico have two types of clusters. Finally, the cities of Chile, Peru, Panama, and Guatemala have only one type of cluster, in addition to Costa Rica, which has only one city in the sample.

**Figure 3:**
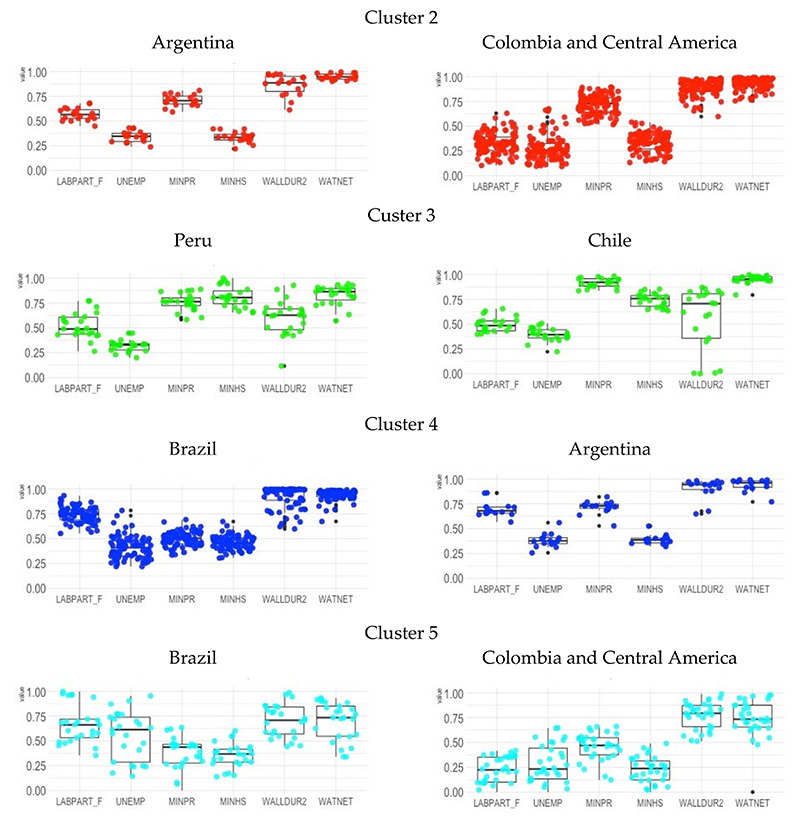
Boxplot of clusters unbundled by cities country. LABPART_F: Labor force participation rate among the female population 15 years or above; UNEMP: The unemployment rate among the total population 15 years or above in the labor force; MINPR: Proportion of the population aged 25 or older who completed primary education or above; MINHS: Proportion of the population aged 25 or older who completed secondary education or above; WALLDUR2: Proportion of dwellings with exterior walls mostly made of brick, stone, concrete, cement, and/or similar materials; WATNET: Proportion of dwellings with water from a public network.

To determine to what extent there are cross-country differences within clusters and to what extent they are similar or different, to understand these regional patterns of typologies better, we disaggregate the clusters by country (except cluster 1, which is located only in Brazil). Considering the regional proximity or distance between the countries, we proceeded with the analysis comparing the two most representative countries or regional blocks of countries of the 4 clusters, as follows: (i) Argentina against Colombia plus Central American countries (Costa Rica, Nicaragua, Mexico, and Colombia), for Cluster 2; (ii) Peru against Chile, for Cluster 3; (iii) Argentina against Brazil, for Cluster 4; and Brazil against Argentina plus Central American countries, for Cluster 5. Results are presented in Figure 3.

The typology of cities defined by Cluster 2 is derived from the distribution of variables with very similar average behavior for the cities of Argentina and Colombia plus Central American countries. The highlighted regional difference occurs in the average female labor force participation, which is higher in Argentine cities than in other cities in the cluster.

The cities defined by the typology of Cluster 3 are mainly placed in three countries (Chile, Peru, and Panama). More than 90% of the cities are in Chile and Peru, so we compare the cities of these two countries. As shown in Figure 3, Chilean cities have lower average values for Secondary education and similar average values for Unemployment, Primary education, Exterior walls, and Water from a public network, in the relations that form the typology of low unemployment rates of these cities.

Ninety-four cities in Argentina and Brazil form the typology defined by Cluster 4, and one city in El Salvador. Because there is only one city from El Salvador, we discuss its main features by considering two non-overlapping groups formed by Brazilian and Argentine cities. The results in Figure 3 show that a higher average value of primary education for Argentine cities within this cluster of cities with the highest female labor force participation highlights the difference between the two groups.

Finally, we consider differences in the typology defined by Cluster 5, formed by cities of different regions of Brazil, Colombia, El Salvador, Guatemala, and Mexico. We separated them into two groups: Brazilian cities and the cities of other countries. This division was motivated by the geographic distribution of cities in Central America and Colombia, and the remaining is distributed across Brazil’s North, Northeast, and South regions. Cluster 5 has the most dispersive distribution and a low average for female labor, as shown in Figure 3. However, the highest averages for female labor participation are in Brazil. The cities also present the highest levels of Primary education and Unemployment to form the typology of low female labor and low education.

In summary, the strategy to identify differences within the clusters allowed us to characterise regional patterns as follows: the low-education cities in the Northeast region of Brazil; low-unemployment cities, Peru and Panama; high primary education cities in Argentina, Chile, Colombia, Costa Rica, Nicaragua, and Mexico; high female labor participation cities with high primary education in Argentina, contrasted to low primary education in Brazil; and low female labor participation and low-education cities in Brazil, Colombia, El Salvador, Guatemala, and Mexico.

## 4 Discussion

This study proposes a socioeconomic typology of cities for Latin America, the most urbanised developing region in the world. We found 5 clusters, which were differentiated based primarily on educational level and labor market conditions (unemployment and labor force participation), since housing quality and water access in the dwelling shows only a few differences across clusters except for Cluster 5. We also observed strong associations of clusters with countries.

Brazil was the country that presented the most significant diversity of typologies.

Despite the presence of some cities of Cluster 1 in the country’s Southeast region, it overwhelmingly contains Cluster 1 cities in the Northeast region and the state of Minas Gerais.

On the other hand, there are similar socioeconomic relations expressed by Cluster 5 among cities in the North and South of Brazil. It could be an unexpected result due to these regions’ social and income differences. However, most of the large cities in the sample of North and Northeast (Cluster 5) are the state capital cities, which are more similar to the South and Southeast region cities regarding socioeconomic conditions. Finally, the cities of the Southeast, the wealthiest region, and some capital cities or more developed cities in the North and Northeast regions of Brazil also shared clustering patterns expressed by the typology of Cluster 4, which is an essential finding in this research.

Regarding the other regional patterns, the Chilean and Peruvian cities were more frequently in Cluster 3, characterized by moderate female labor participation, moderate levels of unemployment, and higher educational level and with a relatively low proportion of housing with durable walls, possibly due to a broader utilisation of wood in the construction of housing in smaller cities (note the broad dispersion of this indicator, especially in Chile (Figure 3), possibly given its resistance to earthquakes in contrast to the high cost of using durable materials in anti-seismic constructions.

In Mexico, Colombia, and Argentina, the city patterns were concentrated in clusters 2, 3, and 5, but up to two typologies per country. However, we highlight the more homogeneous spatial distribution of typologies from clusters 2 and 4 in Argentina, defining a balanced spatial distribution of cities of intermediate socioeconomic status, characterised by either high levels of completed primary education or female labor force participation. Regarding the Central American countries, Guatemala’s cities exhibited Cluster 5, and El Salvador’s cities were Cluster 4 and Cluster 5. Most of Nicaragua and San Jose in Costa Rica were characterised by Cluster 2. Finally, the cities of Panama presented only Cluster 3. Thus, despite the small number of cities in these countries of Central America, it is possible to conclude that cities in Latin America cluster in socioeconomic typologies with regionalised patterns.

The research provided a socio-spatial description of the structure of large urban agglomerations in Latin America. The methodological advances in using census data to find socioeconomic typology can be the basis for more in-depth and detailed studies on Latin American cities in a broad context of structural changes in societies that directly impact the lives of individuals and institutions. There appeared to be a pattern of lower educational levels associated with higher female labor force participation and lower female labor force participation in higher educational level contexts, suggesting that women tend to participate greatly in more informal labor markets [31]. The presence of high female labor participation in Cluster 4, contrasting with the low female labor force in the typology of Cluster 5, also shows that the region needs global policies and specific efforts to decrease the gender gap in the labor market. Heterogeneities in education across clusters also suggests the need for targeted educational policies.

Our study has several limitations. The use of census data for this research constitutes a particular strength. However, the analyses were limited by the domains and variables measured in a similar way across countries. Spatial dependencies and spatial proximities were not considered when the clusters were identified. However, our results reveal significant spatial patterning which is itself of interest and could be further examined in subsequent work. We also did not examine longitudinal changes, the analysis of which is critical to understand dynamics and causal processes. Results also could be influenced by the census availability for different years. Future studies need to expand the analysis by creating or applying other indicators, such as built environment or environmental variables [12]. Future research may link typologies to health or environmental outcomes in cities.

## 5 Conclusions

The recent and current COVID-19 pandemic exposed the many social and economic vulnerabilities of residents in the large urban centers of Latin America [32,33]. Our results highlight important heterogeneities in social and economic indicators across cities and suggest that different typologies can be identified. Future work is needed to understand the drivers and consequences of these typologies. For this reason, we emphasize the importance of interdisciplinary and transdisciplinary research involving a wide range of partnerships between health specialists, urban planners, and social scientists.

Our results also highlight the need to consider the interconnections across domains linked to the Sustainable Development Goals (SDGs). The indicators included in our typologies are connected to SDG 5 (Achieve gender equality and empower all women and girls), SDG 8 (Promote sustained, inclusive, and sustainable economic growth, full productive employment, and decent work for all), SDG 10 (Reduce inequality within and among countries), and SDG 11 (make cities and human settlements inclusive, safe, resilient, and sustainable). Explicit recognition and analysis of these interrelationships are needed to identify the best policy options to make Latin America’s cities an environment with great equity and more opportunity for all populations.

## Supplementary Material

Appendix

## Figures and Tables

**Table 1:** Number of clusters at convergence for different minimum prior probabilities and criteria for convergence.

Minimum Prior Probability	Number of Factors at Convergence by Different Criteria		
	AIC	BIC	ICL
0.00	10	7	7
0.05	10	8	8
0.10	6	6	6
0.15	4	4	4
0.20	3	3	3

## Data Availability

The SALURBAL project welcomes queries from anyone interested in learning more about its dataset and potential access to data. To learn more about SALURBAL’s dataset, visit https://drexel.edu/lac/ or contact the project at salurbal@drexel.edu (accessed on 25 March 2022).
