# Supplementary material for: Socioeconomic Urban Environment in Latin America: Towards a Typology of Cities
Source: Sustainability. Author manuscript; Available in PMC 2024 Dec 9. (PMC7617124; doi:10.3390/su15086380)
Supplement: Appendix [file EMS184312-supplement-Appendix.pdf]

Appendix A

Table A1. Census indicators harmonised in the SALURBAL project by country and year.

Dimension	Subdimension	Variable	Availability Census Data by Year					
			2002	2005	2007	2010	2011	2017
Sanitary conditions	Sanitary conditions	Proportion of households with piped water	CLGT	CONI	PESV	ARBRMXPA	CR	PE
		Proportion of households with piped water access inside the dwelling	CLGT	CONI	PESV	ARBRMXPA	CR	PE
		Proportion of households with water from a public network	CLGT	CONI	PESV	ARBRMXPA	CR	CLPE
		Proportion of households connected to a sewage system of any type	CLGT	CONI	PESV	ARBRMXPA	CR	PE
		Proportion of households connected to a public sewage network	CLGT	CONI	PESV	ARBRMXPA	CR	PE
	Materiality	Proportion of dwellings with exterior walls mostly composed of durable materials	CLGT	CONI	PESV	ARBRMXPA	CR	CLPE
		Proportion of dwellings with exterior walls mostly made of brick, stone, concrete, cement, and/or similar materials (Masonry Walls)	CLGT	CONI	PESV	ARBRMXPA	CR	CLPE
		Proportion of dwellings with exterior walls composed of durable materials	CLGT	CONI	PESV	ARBRMXPA	CR	CLPE
		Proportion of dwellings with finished floors	CLGT	CONI	PESV	AR MXPA	CR	CLPE
	Living conditions	Overcrowding: Proportion of households with more than 3 people per room	CLGT	CO	PESV	ARBRMXPA	CR	PE
		Overcrowding: Proportion of households with more than 2.5 people per bedroom	CLGT	CO	SV	ARBRMXPA	CR	CL
Labour market	Unemployment	The unemployment rate among the total population 15 years or above in the labor force	CLGT	CONI	PESV	ARBRMXPA	CR	CLPE
		The unemployment rate among the male population 15 years or above in the labor force	CLGT	CONI	PESV	ARBRMXPA	CR	CLPE
		The unemployment rate among the female population 15 years or above in the labor force	CLGT	CONI	PESV	ARBRMXPA	CR	CLPE
		The ratio of the unemployment rate among females (15+ years of age) to the unemployment rate among males (15+ years of age)	CLGT	CONI	PESV	ARBRMXPA	CR	CLPE
	Labour participation	The labor force participation rate among the total population 15 years or above	CLGT	CONI	PESV	ARBRMXPA	CR	CLPE
		The labor force participation rate among the male population 15 years or above	CLGT	CONI	PESV	ARBRMXPA	CR	CLPE
		The labor force participation rate among the female population 15 years or above	CLGT	CONI	PESV	ARBRMXPA	CR	CLPE
		The proportion of the total labor force who are female among the population 15 years of age or above	CLGT	CONI	PESV	ARBRMXPA	CR	CLPE
		The ratio of the labor force participation rate among females (15+ years of age) to the labor force participation rate among males (15+years of age)	CLGT	CONI	PESV	ARBRMXPA	CR	CLPE
Education	School attending	Proportion of the population aged 15 to 17 attending school	CLGT	CONI	PESV	ARBRMXPA	CR	PE
		Proportion of the male population aged 15 to 17 attending school	CLGT	CONI	PESV	ARBRMXPA	CR	PE
		Proportion of the female population aged 15 to 17 attending school	CLGT	CONI	PESV	ARBRMXPA	CR	PE
		The ratio of the proportion of the female population aged 15 to 17 attending school to the proportion of the male population aged 15 to 17 attending school	CLGT	CONI	PESV	ARBRMXPA	CR	PE
	Education attainment	Proportion of the population aged 25 or older who completed primary education or above	CLGT	CONI	PESV	ARBRMXPA	CR	CLPE
		Proportion of the male population aged 25 or older who completed primary education or above	CLGT	CONI	PESV	ARBRMXPA	CR	CLPE
		Proportion of the female population aged 25 or older who completed primary education or above	CLGT	CONI	PESV	ARBRMXPA	CR	CLPE
		Proportion of the population aged 25 or older who completed secondary education or above	CLGT	CONI	PESV	ARBRMXPA	CR	CLPE
		Proportion of the male population aged 25 or older who completed secondary education or above	CLGT	CONI	PESV	ARBRMXPA	CR	CLPE
		Proportion of the female population aged 25 or older who completed secondary education or above	CLGT	CONI	PESV	ARBRMXPA	CR	CLPE
		The ratio of the female to the male proportion of the population aged 25 or older who completed secondary education or above	CLGT	CONI	PESV	ARBRMXPA	CR	CLPE
		Proportion of the population aged 25 or older who completed university education or above	CLGT	CONI	PESV	ARBRMXPA	CR	CLPE
		Proportion of the male population aged 25 or older who completed university education or above	CLGT	CONI	PESV	ARBRMXPA	CR	CLPE
		Proportion of the female population aged 25 or older who completed university education or above	CLGT	CONI	PESV	ARBRMXPA	CR	CLPE

Source: SALURBAL Project.

Appendix B

Table A2. Latin American selected cities for the study.

**Argentina, n = 33**										
Bahía Blanca	Comodoro Rivadavia	La Rioja	Rosario	Resistencia	Río Cuarto	Córdoba	San Fernando del Valle de Catamarca	San Miguel de Tucumán-Tafí Viejo	Rawson–Trelew	Bahía Blanca
Mar del Plata	Corrientes	Mendoza	Santa Fe	Santa Rosa-Toay	San Salvador de Jujuy	Formosa	Paraná	Villa Mercedes	Río Gallegos	Mar del Plata
San Nicolás de los Arroyos	Buenos Aires	San Rafael	San Juan	Santiago del Estero- La Banda	Salta	San Carlos de Bariloche	Neuquén-Plottier-Cipolletti	Zarate-Campana	San Luis	San Nicolás de los Arroyos
Tandil	Concordia	Posadas								Tandil
**Brazil, n = 152**										
Rio Branco	Fortaleza	Rondonópolis	Poços de Caldas	Arapongas	Parnaíba	Caxias do Sul	Criciúma	Bragança Paulista	Rio Claro	Parobe
Arapiraca	Juazeiro do Norte	Campo Grande	Pouso Alegre	Cascavel	Teresina	Passo Fundo	Florianópolis	Campinas	São Carlos	Rio das Ostras
Maceió	Sobral	Dourados	Sete Lagoas	Curitiba	Angra dos Reis	Pelotas	Itajaí	Catanduva	São José do Rio Preto	Vitória da Conquista
Macapá	Brasília	Araguari	Teófilo Otoni	Foz do Iguaçu	Araruama	Porto Alegre	Jaraguá do Sul	Franca	São José dos Campos	Cuiabá
Manaus	Cachoeiro de Itapemirim	Barbacena	Uberaba	Guarapuava	Cabo Frio	Rio Grande	Joinville	Guaratinguetá	São Paulo	Patos de Minas
Alagoinhas	Guarapari	Belo Horizonte	Uberlândia	Londrina	Campos dos Goytacazes	Santa Cruz do Sul	Lages	Itapetininga	Sertãozinho	Apucarana
Barreiras	Linhares	Conselheiro Lafaiete	Varginha	Maringá	Macaé	Santa Maria	Araçatuba	Jaú	Sorocaba	Vitória de Santo Antão
Feira de Santana	Vitória	Divinópolis	Belém	Paranaguá	Nova Friburgo	Uruguaiana	Araraquara	Jundiaí	Tatuí	Natal
Ilhéus	Anápolis	Governador Valadares	Castanhal	Ponta Grossa	Petrópolis	Ji-Paraná	Araras	Limeira	Taubaté	Chapecó
Itabuna	Goiânia	Ipatinga	Marabá	Toledo	Resende	Porto Velho	Atibaia	Marília	Aracaju	Botucatu
Jequié	Rio Verde	Itabira	Parauapebas	Caruaru	Rio de Janeiro	Boa Vista	Santos	Mogi Guaçu	Araguaína	Ribeirão Preto
Porto Seguro	Caxias	Juiz de Fora	Santarém	Garanhuns	Teresópolis	Balneário Camboriú	Barretos	Ourinhos	Palmas	Caraguatatuba
Salvador	Imperatriz	Montes Claros	Campina Grande	Petrolina	Volta Redonda	Blumenau	Bauru	Piracicaba	Tubarao	
Teixeira de Freitas	São Luís	Passos	João Pessoa	Recife	Mossoró	Brusque	Birigui	Presidente Prudente	Bento Goncalves	
**Chile, n = 21**										
Arica	La Serena-Coquimbo	Rancagua	Los Ángeles	Copiapó	Punta Arenas	Calama	Valdivia	Quillota	Concepción	Antofagasta
Iquique	Valparaíso-Viña del Mar	Talca	Temuco	Santiago de Chile	Osorno	San Antonio	Curicó	Puerto Montt	Chillán	
**Colombia, n = 35**										
Apartadó	Florencia	Bogotá	Cúcuta	Ibagué	Duitama	Manizales	Tunja	Palmira	Guadalajara de Buga	Cartago
Medellín	Yopal	Neiva	Armenia	Buenaventura	Girardot	Montería	Quibdó	Sogamoso	Sincelejo	Barrancabermeja
Barranquilla	Popayán	Riohacha	Pereira	Cali	Fusagasugá	Pasto	Villavicencio	Tuluá	Bucaramanga	Santa Marta
									Cartagena	Valledupar
**Peru, n = 23**										
Chimbote	Cusco	Huancayo	Piura	Tacna	Cajamarca	Ayacucho	Tarapoto	Puno	Iquitos	Trujillo
Huaraz	Huánuco	Chiclayo	Sullana	Tumbes	Pisco	Ica	Pucallpa	Juliaca	Lima	Chincha Alta
										Arequipa
**Mexico, n = 92**										
Ensenada	Monclova	Uriangato	Zamora de Hidalgo	Playa del Carmen	Matamoros	Ciudad Acuña	Tuxtla Gutiérrez	Tapachula	San Cristóbal de las Casas	Hidalgo del Parral
Mexicali	Piedras Negras	Salamanca	Cuautla	Culiacán	Nuevo Laredo	León	Irapuato	Guanajuato	Celaya	Iguala
Tijuana	Saltillo	San Francisco del Rincón	Cuernavaca	Los Mochis	Reynosa	Uruapan	Morelia	La Piedad	Toluca	Tianguistenco
La Paz	Torreón	Pachuca	Tepic	Mazatlán	Tampico	Chetumal	Cancún	San Juan del Río	Querétaro	Teziutlán
Campeche	Colima	Tula de Allende	Monterrey	Ciudad Valles	Tlaxcala	Ciudad Victoria	Villahermosa	San Luis Río Colorado	Nogales	Navojoa
Ciudad del Carmen	Manzanillo	Tulancingo	Oaxaca	Rioverde	Acayucan	Fresnillo	Mérida	Xalapa-Enríquez	Veracruz	Poza Rica de Hidalgo
Chihuahua	Tecomán	Guadalajara	San Juan Bautista Tuxtepec	San Luis Potosí	Coatzacoalcos	Aguascaliente	Minatitlán	Guaymas	Puebla	Puerto Vallarta
Ciudad Juárez	Durango	Ocotlán	Santo Domingo Tehuantepec	Ciudad Obregón	Córdoba	Zacatecas	Orizaba	Hermosillo	Tehuacán	Ciudad de México
							Cuauhtémoc	Delicias	Chilpancingo	Acapulco
**Costa Rica, n = 1**		**El Salvador, n = 3**		**Guatemala, n = 3**		**Nicaragua, n = 5**			**Panama, n = 3**	
San José		San Miguel		Escuintla		Chinandega	Managua		Panama City	
		San Salvador		Ciudad de Guatemala		Estelí	Masaya		Colon	
		Santa Ana		Quetzaltenango		León			David	

Source: SALURBAL Project.

Appendix C

Table A3. Exploratory factor analysis of the city level social environment indicators—factor loadings.

Sets Variables Loading onto Each Factor		Factor 1	Factor 2	Factor 3	Factor 4	Factor 5	Communality
Labor force participation	Ratio of female to male unemployment	0.774		−0.227	0.278		0.74
	Labor force participation (female and male)	0.917			−0.228	0.219	0.94
	**Female labor force participation**	0.964	0.135			0.205	**0.99**
	Male labor force participation	0.509	−0.260	0.148	−0.491	0.219	0.63
	Proportion of the total labor force that is female	0.565	0.195	−0.362		0.455	0.70
	Ratio of female to male labor force participation	0.917	0.251			0.153	0.94
	Ratio of the female to the male proportion of the population aged 25 or older who completed secondary education or above	0.490	−0.208	−0.229	0.347		0.46
Unemployment	**Unemployment rate (female and male)**	0.115	−0.128		0.981		**0.99**
	Male unemployment rate	−0.246	−0.163		0.915		0.93
	Female unemployment rate	0.399		−0.180	0.868		0.95
Primary education	Proportion of the population aged 25 or older who completed primary education or above	−0.258	0.219	0.799			0.79
	**Proportion of the male population aged 25 or older who completed primary education or above**	−0.221	0.286	0.915			**0.97**
	Proportion of the female population aged 25 or older who completed primary education or above	−0.160	0.205	0.931			0.93
Secondary and higher education	Proportion of the female population aged 25 or older who completed secondary education or above	0.233	0.611	0.240		−0.487	0.73
	Proportion of the population aged 25 or older who completed secondary education or above	0.223	0.716	0.507	0.184	−0.316	**0.95**
	Proportion of the male population aged 25 or older who completed secondary education or above	0.399	0.651	0.461	0.297	−0.282	0.96
	Proportion of the population aged 25 or older who completed university education or above		0.828		−0.275	0.107	0.78
	**Proportion of the male population aged 25 or older who completed university education or above**	−0.247	0.881	0.197	−0.206	0.250	**0.98**
	Proportion of the female population aged 25 or older who completed university education or above		0.906			0.269	0.90
Masonry Walls	Proportion of dwellings with exterior walls mostly composed of durable materials		0.211		−0.127	0.204	0.10
	**Proportion of dwellings with exterior walls mostly made of masonry**	0.180				0.546	**0.33**
	Proportion of dwellings with exterior walls composed of durable materials	0.129	0.106			0.487	0.27
Water public network	Proportion of dwellings with water from a public network			0.416		0.185	**0.22**
% common variance explained		20.9	17.9	14.8	14.7	6.9	–

Note: In bold is the indicator with the highest communality per factor and in italic the selected name to represent the set.

Table A4. Summary statistics of 6 selected (final) variables.

Variable	Description	Mean	Median	Min	Max	Skewness	Kurtosis
Female labor force	Labor force participation rate among the female population 15 years or above	49.04	49.57	26.16	70.17	−0.15	−0.74
Unemployment rate	The unemployment rate among the total population 15 years or above in labor force	7.03	6.53	0.55	17.47	0.79	0.59
Primary education	Proportion of population aged 25 or older who completed primary education or above	73.32	73.23	43.30	93.63	−0.19	−0.51
Secondary education	Proportion of the population aged 25 or older who completed secondary education or above	40.29	38.57	13.04	79.08	79.08	1.12
Water public network	Proportion of dwellings with water from a public network	89.04	92.86	5.77	99.51	−2.61	9.96
Masonry Walls	Proportion of dwellings with exterior walls mostly made of brick, stone, concrete, cement, and/or similar materials	87.82	92.80	19.15	99.91	−2.03	5.47

Source: Elaborated by the authors based on the research results.
